# The Impact of Doctor of Nursing Practice Education on Career Advancement and Professional Satisfaction: A Scoping Review

**DOI:** 10.1111/jan.16976

**Published:** 2025-04-16

**Authors:** Tuba Sengul, Seda Sarikose, Violeta Lopez, Holly Kirkland‐Kyhn

**Affiliations:** ^1^ Koç University School of Nursing Istanbul Türkiye; ^2^ School of Nursing, Midwifery and Social Sciences Central Queensland University Rockhampton Australia; ^3^ School of Nursing and Allied Medical Sciences Holy Angel University Angeles Philippines; ^4^ The Betty Irene Moore School of Nursing at UC Davis Sacramento California USA

**Keywords:** career pathways, doctoral education, mentorship, nursing, nursing practice, satisfaction

## Abstract

**Aim:**

To explore the impact of Doctor of Nursing Practice (DNP) education on career advancement, job satisfaction, leadership competencies and contributions to healthcare systems.

**Design:**

The study utilised a scoping review methodology based on Arksey and O'Malley's (2005) framework.

**Methods:**

The search strategy was developed with an academic librarian to ensure thoroughness and relevance. Seven databases were searched using MesH terms. Inclusion criteria focused on peer‐reviewed studies examining DNP education's influence on career advancement, job satisfaction and leadership. Thematic analysis was used to identify patterns and themes.

**Data Sources:**

Studies were selected based on their focus on DNP‐prepared nurses, nursing faculty or advanced practice nursing students in healthcare or academic settings, published between 2004 and 2024.

**Results:**

Twenty‐one studies met the inclusion criteria, highlighting DNP education's role in fostering leadership, professional development and evidence‐based practice. Thematic analysis revealed the benefits of being a DNP graduate include contribution to professional development, contribution to leadership and contribution to the practice environment. The challenges to DNP graduates include underrecognition of competencies, high educational costs and limited academic opportunities that were also identified.

**Conclusions:**

DNP education contributes to individual and professional growth, leadership development and healthcare system improvements. However, barriers such as financial constraints and inadequate recognition of DNP competencies must be addressed to maximise the impact of this educational model.

**Implications for the Profession and/or Patient Care:**

DNP education empowers nurses to lead healthcare innovations, enhance patient care quality and reduce disparities in health outcomes. Strengthening financial and systemic support for DNP graduates is essential for sustaining these contributions.

**Impact:**

DNP education is a transformative force in nursing, offering significant opportunities for leadership development and healthcare advancements. Aligning DNP programmes with evolving global healthcare challenges can further strengthen their impact on the profession and patient care.

**Reporting Methods:**

PRISMA‐ScR guidelines were followed.

## 1 Introduction

Doctoral education in nursing is a powerful tool for career advancement, professional satisfaction and leadership in healthcare systems (Van Dongen et al. 2024; Beeber, Johnson, and Lee 2019; Beeber, Palmer, et al. 2019). Doctoral programmes equip nurses with advanced knowledge, research skills and leadership competencies, enabling them to address the complexities of modern healthcare systems (Kesten et al. 2022; Chavez et al. 2019). This education, offered through practice‐focused Doctor of Nursing Practice (DNP) or research‐based Doctor of Philosophy (PhD) programmes, functions as a bridge that integrates theory and practice (American Association of Colleges of Nursing 2022a, 2022b; Fang and Bednash 2017). Globally, there is significant variation in the structure and accessibility of doctoral nursing education. While the United States, Canada, Finland, the United Kingdom and Australia have well‐established doctoral programmes integrating clinical practice and research, other regions face significant challenges in implementation (Kim et al. 2022). In contrast, regions like Saudi Arabia, Jordan and Ghana encounter obstacles related to resource constraints and a lack of standardised doctoral pathways (Anthony et al. 2020). These countries often face challenges such as faculty shortages, funding issues and the need for international collaboration to meet global standards (Anthony et al. 2020; Kim et al. 2022). The development of doctoral programmes in these regions is often influenced by the need to address specific health issues, such as non‐communicable diseases, and to align with international standards (Anthony et al. 2020; European Federation of Nurses Associations 2022; Kim et al. 2022). Furthermore, many countries, including Germany, Türkiye and several Eastern European nations, do not offer DNP programmes, thereby limiting clinical leadership pathways for advanced practice nurses (De Raeve et al. 2024; Semerci et al. 2024). The absence of these programmes restricts nurses' ability to lead healthcare transformations, implement evidence‐based practices in clinical settings and contribute to policy development at the same level as their counterparts in countries with established DNP programs (American Association of Colleges of Nursing 2022a, 2022b; European Federation of Nurses Associations 2022; Kim et al. 2022). Despite these discrepancies, efforts to globalise doctoral nursing education through international collaborations and online learning platforms are increasing (Fang and Bednash 2017; Moran et al. 2023).

In recent years, the demand for nurses with doctoral‐level education has grown, with healthcare organisations expecting these professionals to shape policies, lead interdisciplinary teams and drive evidence‐based practices (Brown et al. 2020; Moran et al. 2023). Additionally, doctoral education enhances personal and professional growth, enabling nurses to align their career goals with professional values (Giordano et al. 2021; Chavez et al. 2019). Doctoral education directly impacts career trajectories by offering advanced nursing roles in academic and clinical settings (Dobrowolska et al. 2021). PhD programmes foster expertise in scientific research and theoretical framework development, while DNP programmes emphasise clinical practice and systems leadership (Brown et al. 2020; Dobrowolska et al. 2021). These two models contribute uniquely to career development. PhD graduates often advance in research and education, whereas DNP graduates pursue leadership positions that drive innovative practices in healthcare (McCauley et al. 2020). However, developing hybrid models that train nurses with theoretical and practical expertise is essential to meet modern healthcare systems' dynamic needs (McCauley et al. 2020; American Association of Colleges of Nursing 2022a, 2022b).

Doctoral education also significantly impacts professional satisfaction by enabling nurses to engage in meaningful work (Kesten et al. 2022). Research indicates doctoral‐prepared nurses report higher satisfaction levels due to opportunities to engage in advanced practices, shape organisational policies and drive system‐wide improvements (Kesten et al. 2022; Beeber, Johnson, and Lee 2019; Beeber, Palmer, et al. 2019). Moreover, doctoral programmes foster critical thinking, autonomy, and collaboration—key skills contributing to professional fulfilment (Beeber, Johnson, and Lee 2019; Beeber, Palmer, et al. 2019). However, satisfaction levels may vary depending on the alignment of education with workplace roles. For instance, DNP graduates working in positions that underutilise their advanced skills often report lower satisfaction levels (Chavez et al. 2019). Despite its benefits, access to doctoral education is not equitable. Socioeconomic barriers, underrepresentation of minority groups and geographic limitations hinder many individuals from enrolling in such programmes (Avery‐Desmarais et al. 2021). This limits individual career growth and results in a lack of diverse perspectives in the nursing profession (Fang and Bednash 2017). Innovative strategies such as scholarship programmes, mentorship opportunities and flexible learning models are critical to overcoming these barriers (Fang and Bednash 2017; Weaver et al. 2023). Increasing access to doctoral programmes, particularly for underrepresented groups, is a crucial step towards promoting equity in healthcare (Moran et al. 2023).

Doctoral education is a cornerstone of the nursing profession's future. Integrating technological innovations, modern curricula and equitable policies presents significant opportunities for preparing nurse leaders (Avery‐Desmarais et al. 2021; Beeber, Johnson, and Lee 2019; Beeber, Palmer, et al. 2019; Kesten et al. 2022). In the future, priorities should include fostering interdisciplinary collaborations, developing hybrid doctoral models, and creating leadership programmes that address global healthcare needs (Van Dongen et al. 2024; Chavez et al. 2019; Fang and Bednash 2017). Evaluating the impacts of doctoral education continuously, adopting innovative approaches and implementing strategies to enhance individual and professional satisfaction will drive the nursing profession forward (American Association of Colleges of Nursing 2022a, 2022b; Fang and Bednash 2017; Giordano et al. 2021). This study aimed to comprehensively evaluate the effects of doctoral education on career advancement, leadership capacities and professional satisfaction among nurses, addressing existing knowledge gaps in these areas.

## 2 The Review

The review applied Arksey and O'Malley's (2005) methodological framework for scoping studies, and findings were categorised into themes using a structured approach for thematic analysis (Braun and Clarke 2006).

### 2.1 Aims

This scoping review explored the impact of DNP education on career advancement and job satisfaction, highlighting how DNP education contributes to leadership, professional development and practice improvements in nursing.

### 2.2 Research Questions

- What career advancement opportunities are associated with completing a DNP programme?
- How does DNP education influence job satisfaction among advanced practice nurses and nursing leaders?
- What challenges do DNP graduates face in achieving career growth and satisfaction?
- How does DNP education prepare nurses for leadership roles and impact their contributions to healthcare systems?
- What strategies or resources can enhance DNP‐prepared nurses' career satisfaction and professional outcomes?

## 3 Methods

### 3.1 Design

This scoping review used the methodological framework that Arksey and O'Malley (2005) outlined to explore the impact of DNP education on career advancement and job satisfaction. The framework provides a systematic approach consisting of five key steps: defining the research questions, identifying and selecting relevant studies, screening and including studies for review, organising and charting the data, and finally, synthesising and reporting the findings. These steps ensured a rigorous and structured approach throughout the review process. To analyse the data, Clarke and Braun's (2017) six‐step thematic analysis framework was applied. This process involved familiarising myself with the data, generating initial codes, identifying and reviewing themes and organising the results into meaningful categories. This combination of frameworks allowed for an in‐depth exploration of patterns and themes within the selected studies. In addition to Arksey and O'Malley's (2005) guidelines, this review followed the Preferred Reporting Items for Systematic Reviews and Meta‐Analysis extension for Scoping Reviews (PRISMA‐ScR) developed by Tricco et al. (2018). This scoping review also incorporates the evidence‐based checklist for improving scoping review quality (Cooper et al. 2019) to systematically map existing knowledge on the research topic.

### 3.2 Search Methods

The study keywords were developed through Medical Subject Headings (MeSH) and guided by the PCC (Population, Concept, Context) framework (Pollock et al. 2023). This approach ensured a comprehensive literature search aligned with the objectives of the scoping review.

#### 3.2.1 Population (P)

- Nurses who have completed or are currently enrolled in DNP programmes.
- Nursing educators and leaders involved in advanced practice nursing education.

#### 3.2.2 Concept (C)

Impact of DNP education on the following:

- Career advancement (e.g., promotions, career trajectories).
- Professional satisfaction (e.g., job satisfaction, fulfilment).
- Leadership roles (e.g., administrative or managerial positions).
- Professional development (e.g., skill enhancement, contributions to nursing practice).
- Includes comparisons of outcomes among DNP‐prepared nurses versus those holding other nursing or healthcare‐related doctoral degrees and examinations of career trajectories for DNP‐prepared nurses compared to non‐doctorally prepared nurses.

#### 3.2.3 Context (C)

Settings: Healthcare environments, academic institutions or professional organisations where DNP‐prepared nurses are employed or where DNP programmes are offered.

Timeframe: Studies published from 2004 onwards reflecting the formal establishment and evolution of DNP programmes.

Study Designs: Randomised controlled trials, longitudinal studies, cross‐sectional studies, descriptive research, qualitative studies and mixed‐methods research that focus on the impact of DNP education on career outcomes and satisfaction.

All peer‐reviewed, full‐text articles published in English during the specified timeframe were considered for inclusion. This broader inclusion strategy aligns with scoping review methods, which aim to map existing evidence comprehensively.

### 3.3 Information Sources

The search strategy was developed with an academic librarian to ensure a systematic and thorough literature review. This scoping review followed the Preferred Reporting Items for Systematic Reviews and Meta‐Analyses extension for Scoping Reviews (PRISMA‐ScR) checklist (Supplementary File 1). Articles were retrieved from seven databases: PubMed, CINAHL, Scopus, Web of Science, Medline (OVID), ProQuest Dissertations & Theses Global, and ScienceDirect. Searches were conducted on “Advanced Practice Nursing,” “Career Advancement,” “Job Satisfaction,” “Professional Satisfaction,” “Leadership in Nursing,” “Professional Development,” “DNP‐prepared Nurses,” “Nursing Career Growth,” and “Doctoral Education in Nursing.” Relevant subject headings (e.g., MeSH terms) were incorporated into each database to optimise search results. Boolean operators (‘and’, ‘or’) were employed to refine the strategy and ensure comprehensive retrieval. All identified records were imported into Covidence software for systematic screening, de‐duplication and review. Inclusion criteria focused on studies examining the impact of DNP education on career advancement and satisfaction, while exclusion criteria removed articles unrelated to the nursing profession or doctoral education. An academic librarian was consulted throughout the process to validate and optimise the search strategy, and additional searches were performed to capture any key concepts or critical literature that might have been missed.

### 3.4 Inclusion and Exclusion Criteria

#### 3.4.1 Studies Were Included Based on the Following Criteria

Peer‐reviewed empirical studies with quantitative, qualitative or mixed‐methods research designs, systematic reviews or scoping reviews. Research focusing on the impact of DNP education on career advancement, professional satisfaction, leadership roles or professional development was included. Participants included DNP‐prepared nurses, DNP students or nursing faculty involved in DNP education, as well as full‐text articles published in peer‐reviewed journals in English. Articles published between 1 January 2004 and 30 November 2024, reflect the establishment and evolution of DNP programmes. Studies report specific outcomes related to career advancement, job satisfaction or leadership among DNP‐prepared professionals.

### 3.5 Exclusion Criteria

#### 3.5.1 The Following Types of Studies Were Excluded

Studies focusing on doctoral education unrelated to DNP programmes (e.g., PhD or EdD); research addressing general aspects of nursing education without specific relevance to DNP education; articles published in predatory journals, protocols or those lacking sufficient results; non‐English studies, non‐empirical studies, commentaries, editorial letters and unpublished dissertations or theses; studies discussing DNP roles need clear links to career advancement, job satisfaction or leadership outcomes.

### 3.6 Search Outcome

**FIGURE 1:**
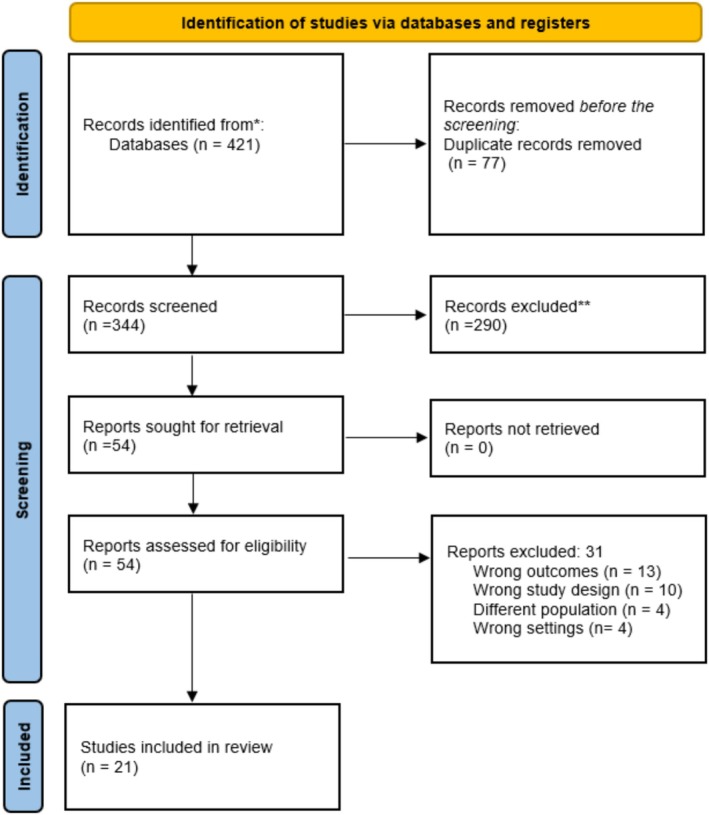
PRISMA flowchart.

The initial search identified 421 records from database sources. After removing 77 duplicate records before screening, 344 were screened based on predefined exclusion criteria. Of these, 290 records were excluded during the screening phase and 54 reports were sought for retrieval, with none being excluded due to retrieval issues. These reports were assessed for eligibility, resulting in the exclusion of 31 studies for reasons including wrong outcomes (*n* = 13), wrong study design (*n* = 10), different population (*n* = 4) and wrong settings (*n* = 4). Ultimately, 21 studies met the inclusion criteria and were incorporated into the scoping review. Figure 1 provides a detailed overview of the identification, screening and inclusion process following the PRISMA framework.

### 3.7 Data Abstraction

The study selection process used Covidence, a systematic review management tool, to ensure an organised and transparent workflow. Four reviewers (VL, HK, TS and SS) independently screened the titles and abstracts of all identified studies based on the predefined inclusion and exclusion criteria. Covidence facilitated the identification and management of conflicts during the initial screening process. In cases where conflicts arose, the third reviewer (VL) resolved disagreements to achieve consensus. After the title and abstract screening phase, full‐text articles were reviewed collaboratively by all three reviewers to ensure alignment with the inclusion criteria. Utilising a templated spreadsheet, the primary researcher reviewed each study for the preliminary charting. Then, the other researchers jointly considered the final data charting to agree on the data to be extracted (Table 1). This iterative process allowed for a rigorous and systematic approach to study selection, minimising bias and maintaining methodological consistency.

**TABLE 1:** Characteristics of included studies for systematic review (*N* = 21).

Title, Authors (year)	Country	Population	Design	Aim	Data collection tools	Outcomes	Perspective
The role of Doctor of Nursing Practice‐prepared nurses in practice settings, Beeber et al. (2019b)	US	DNP Graduates (*n* = 155) DNP programme director (*n* = 34)	Descriptive exploratory‐mixed methods	To examine the settings in which DNPs were employed outside of academia, the positions into which they were hired and their roles in their organisations.	Online survey tool (descriptive information about the programs) in which DNP‐prepared nurses are educated (i.e., programme leadership, modality and profit status) DNP programme director online survey	DNP‐prepared nurses primarily serve in APRN roles (NP, CRNA, CNM) or management positions (chief nurse, quality of care director) DNP nurses contribute to leadership and quality improvement projects, serving as role models in the workplace. DNP‐prepared nurses take on leadership roles that drive organisational change processes. DNP education equips nurses with skills in data analysis, systems‐level thinking, and policy development DNP‐prepared nurses improve patient and organisational outcomes through projects such as reducing infection rates or enhancing patient self‐care.	Career Advancement Job satisfaction Leadership Professional development Practice improvements
Voices of chief nursing executives informing a doctor of nursing practice program, Embree et al. (2018)	US	Chief nurse executives' (CNEs) (*n* = 10)	Qualitative	To describe the business case framework used to guide doctor of nursing practice (DNP) programme enhancements and to discuss methods used to gain chief nurse executives' (CNEs) perspectives for desired curricular and experiential content for doctor of nursing practice nurses in healthcare system executive roles	Interview questions (e CNE's views of essential knowledge, skills and abilities needed to fulfil systems‐level healthcare leadership roles)	DNP‐prepared nurse leaders offer significant opportunities for career advancement in healthcare systems. DNP‐prepared nurses excel in relationship management and communication, contributing to job satisfaction and organisational success DNP education equips nurses with systems‐level thinking, relationship management, and leadership skills DNP education supports professional growth but requires personal development, quality improvement, and data management enhancements to align with evolving healthcare needs DNP nurse leaders contribute to patient safety and population health management through evidence‐based initiatives	Career Advancement Job satisfaction Leadership Professional development Practice improvements
A course‐based approach to the Doctor of Nursing Practice Project supporting student growth from concept to completion, Fitzgerald and McNulty (2017)	US	Post–master's degree (*n* = 6) and Post‐baccalaureate degree students (*n* = 34)	Course‐based approach	To describe a course‐based approach to the doctor of nursing practice project in which students work in groups of 8–12 with a faculty member to complete individual final projects that require a minimum of 360 practicum hours in three‐semester‐long courses	—	The course‐based DNP project approach equips students with advanced leadership and evidence‐based practice skills, fostering career advancement. Faculty‐guided projects prepare graduates for organisational leadership roles and enhance their professional trajectories. DNP projects address organisational needs through quality improvement initiatives and organisational assessments. Outcomes are shared through presentations and publications, driving meaningful improvements in healthcare delivery	Career advancement Job satisfaction Leadership Professional development Practice improvements
Doctor of nursing practice students' perceptions of professional change through the DNP program, Giardino and Hickey (2020)	US	Post master's DNP Students (*n* = 42)	Thematic analysis study	To describe and discuss perceptions of graduating post master's DNP students regarding personal and professional development that occurred during the DNP programme to identify how DNP graduates saw themselves change and grow during the course of the DNP programme	Reflection paper assignment	The DNP programme prepares graduates for advanced leadership roles, equipping them to advocate for policies, implement evidence‐based improvements, and confidently lead organisational change DNP graduates described the DNP journey as transformative, fostering confidence, professional identity, and satisfaction through meaningful contributions to healthcare DNP education builds leadership skills such as systems thinking, quality improvement, and team collaboration, empowering graduates to address complex healthcare challenges effectively DNP graduates excel in translating evidence into practice, leading quality initiatives, and implementing systemic changes to improve patient care and organisational efficiency	Career advancement Job satisfaction Leadership Professional development Practice improvements
Inconsistency in Faculty and student Perceptions of DNP and PhD Leader Scholarly Activity, Jenkins and McCauley (2021)	US	Doctoral nursing faculty (*n* = 18), DNP or PhD student (*n* = 10)	Action research project	To quantify faculty and student perceptions of scholarly activities for doctor of nursing practice (DNP) and PhD leaders.	Discussion and education sessions with Faculty and online and paper survey	Clear delineation of practice and research objectives for DNP and PhD nursing leaders. Collaborative course design, including interprofessional education experiences, was implemented to bridge gaps and foster career advancement opportunities for DNP and PhD students DNP and PhD students valued joint learning experiences that clarified their roles and promoted mutual understanding of research and quality improvement. Integrated courses on systems thinking and inquiry methods strengthened leadership skills, fostering collaboration and preparing students to address healthcare challenges. Shared learning experiences enhanced understanding of quality improvement and evidence‐based practices, advancing healthcare delivery through improved doctoral education	Career advancement Leadership Professional development Practice improvements
Perceptions of Nurse Practitioners and Physician Assistants/Associates Towards the Concept of Developing an Advanced Practice Postgraduate Residency/Fellowship Program at a Large Academic Medical Center, Kidd and McCauley (2024)	US	Nurse practitioners (NPs) and physician assistants/associates (PAs) (*n* = 126)	Cross‐sectional single‐large academic medical centre (AMC) study	To examine the attitudes of NP and PA career staff towards formalised APP postgraduate training at a large academic medical centre (AMC)	An anonymous electronic survey	Benefits of APP residency/fellowship programme 1. Enhance specialty‐specific knowledge and training, improve medical decision‐making and promote professional development 2. Interest in serving as clinical preceptors for such programmes, indicating potential for career growth opportunities within the organisation 3. Foster professional growth and improving clinical education 4. Supported the value of accreditation for these programs, particularly those working in medical specialties	Career advancement Job satisfaction Leadership Professional development
Practice environment, independence, and roles among DNP‐ and MSN‐prepared Primary Care Nurse practitioners, Martsolf and Auerbach (2021)	US	NPs (*n* = 1148)	Cross‐sectional design	To compare practice environment, independence and roles among DNP‐ and MSN‐prepared primary care NPs	Nurse Practitioner‐Primary Care Organisational Climate Questionnaire (NP‐PCOCQ)	1. DNP‐prepared NPs allocated more time to leadership activities 2. DNP‐prepared NPs reported slightly more favourable practice environments and better relationships with physicians 3. DNP‐prepared NPs spent less time in direct clinical care and more time in practice leadership roles 4. DNP‐prepared NPs on patient outcomes and care quality	Career advancement Job satisfaction Leadership Professional development
Celebrating over 20 years: Outcomes from the first doctor of nursing practice program, Melander et al. (2023)	US	Nurses graduated from the UKCON DNP programme (*n* = 348)	A cross‐sectional mixed methods	To delineate the achievements, outcomes and perception of the impact of the Kentucky College of Nursing DNP programme graduates and assess the correlations of specific “perception of impact” variables	Multiple open‐ended questions and an online survey	1. DNP graduates received at least one promotion post‐degree, with promotions increasing over time 2. Graduates served in varied roles: in practice, leadership and support positions 3. Graduates reported satisfaction with professional growth and leadership opportunities 4. Graduates frequently led quality improvement initiatives, organisational change projects and new programme development 5. Graduates reported significant growth in evidence‐based practice, quality improvement and interprofessional collaboration 6. DNP education enabled them to enhance patient care quality, implement evidence‐based initiatives and address healthcare system challenges	Career advancement Job satisfaction Leadership Professional development Practice improvements
DNPs' labour participation, activities, and reports of degree contributions, Minnick et al. (2019)	US	DNP‐prepared members: the American Organisation of Nurse Executives (AONE), the American Association of Nurse Practitioners (AANP) and the American Association of Nurse Anaesthetists (AANA) (*n* = 1308)	Descriptive survey design methodology	To (1) describe the labour participation and employment of DNP graduates who are members of at least one of three professional nursing organisations (2) describe these DNPs' reports of work activities since graduation and (3) describe their reports of the personal contribution of degree attainment and belief about the need for every APRN to obtain the degree	Survey DNP project experience, scholarship before and after earning a DNP, employment since earning the DNP and views about the contribution and necessity of the degree, DNP Essentials, organisational change, quality improvement, evidence‐based practice, leadership and policy setting	DNP graduates were employed, with nearly all in roles requiring nursing expertise DNP titles as CRNAs and nurse practitioners, educational and administrative roles. While the DNP was not often required for most positions DNP education significantly enhanced leadership, quality improvement, and evidence‐based practice skills DNP‐ graduates had limited publications beyond their DNP projects, but those in faculty and administrative roles were more productive Leadership in organisational change and quality improvement projects was common, particularly among those with over five years of experience The degree supported job promotions, future career flexibility and personal satisfaction, though some respondents cited financial burdens as drawbacks	Career advancement Job satisfaction, Leadership, Professional development
Academic‐practice partnership for Doctor of Nursing Practice in a Large Medical Center, (Prado‐Inzerillo et al. (2023)	US	DNP graduates (*n* = 32)	Descriptive study	To explore the DNP graduates' and students' narratives regarding the benefits to which doctoral education impacted their leadership skills, practice changes, professional growth and initiation of community‐based health projects	AONL Delphi survey	DNP graduates reported increased professional satisfaction, enhanced confidence, critical thinking abilities, networking opportunities, and the use of evidence‐based leadership practices Graduates recognised the programme's contribution to their professional growth and career advancement Graduates' scholarly projects primarily focused on leadership within healthcare systems, including transformational leadership, staff engagement, accountability, relational leadership models and interprofessional collaboration The doctoral education fostered leadership skills and practice changes while driving initiatives like community‐based health projects and innovative leadership development programmes	Career advancement Job satisfaction Leadership Professional development Practice improvements
Enhancing nurse leadership through a cohort‐based model, Robinson (2023)	US	DNP nursing students (*n* = 12)	Cohort‐Based Model	To assess programme implementation, its impact on the participating nurses, and the project's effects on the Health Care Network	Survey‐open enden questions (reflect on the professional impact of the DNP programme, programme implementation, impact on the participating nurses and the project's effects on the Health Care Network)	1. DNP programme participants transition into leadership roles 2. The programme also provides professional incentives that support nurse retention 3. Many participants expressed enthusiasm for continued professional growth, including enrolling in advanced leadership academies and showcasing how the programme motivates career progression 4. DNP programs improve morale through mentorship and a supportive cohort culture 5 DNP education equips nurses with leadership skills to act as change agents, mentorship and management 6 Graduates make a lasting impact as role models for excellence in nursing care, inspiring other organisations 7 DNP programmes empower nurses to lead, innovate, and elevate the profession globally, ensuring improved patient care and organisational success	Career advancement Job satisfaction Leadership Professional development Practice improvements
Applying complexity science as a DNP quantum leader Root et al. (2020)	US	4 unique clinical/organisational examples	Observational Studies	To present four unique clinical/organisational examples that illustrate the applications of complexity and quantum leadership theories in practice changes	Clinical/organisational examples that illustrate the applications of complexity and quantum leadership theories in practice changes	DNP training equips leaders with the tools to create dynamic teams, achieve professional fulfilment, and adopt inclusive leadership practices DNP education prepares leaders to navigate and transform organisational systems	Leadership Professional development Practice improvements
Why pursue a doctorate? Findings From a study of doctorally prepared RNs in an integrated healthcare system, Rosenfeld (2024)	US	RNs at a healthcare system who have completed doctorates (*n* = 93)	Descriptive study	To examine the motivations and perceptions of RNs with completed doctorates in an integrated healthcare system	30‐item survey (motivations for pursuing doctoral education; and 3) perceptions of value and benefits of doctoral education in current and future employment)	DNPs pursue their degrees to fulfil personal and professional goals, with a notable emphasis on clinical practice focus The structured design of DNP programs, including asynchronous online coursework and shorter durations, further motivates candidates DNP degree underscores its contribution to professional development and practice improvements Graduates reported enhanced skills and knowledge, making them more effective in their roles and improving healthcare outcomes for patients and providers A DNP degree provides career competitiveness opportunities and elevates organisational status and respect Lower access to positions typically associated with research and academic collaboration than PhDs	Career advancement Job satisfaction Leadership Professional development Practice improvements
An enhanced cultural competence curriculum and changes in transcultural self‐efficacy in Doctor of Nursing Practice students, Singleton et al. (2017)	US	89 DNP students who began and completed the programme	Pre‐post paired *t*‐test, non‐experimental design	To assess the effectiveness of enhancing cultural competence across a new DNP curriculum on TSE perceptions in DNP‐family nurse practitioner (FNP) students	The 83‐item tool that ‘measures and evaluates students’ confidence in performing general transcultural nursing skills among the diverse population	DNP education contributes to leadership, professional development and practice improvements in nursing DNP programmes prepare graduates to excel in leadership roles and advance nursing practice Institutional and professional support to sustain educational initiatives that promote culturally competent care and career advancement for DNP graduates	Career advancement Professional development Practice improvements
Scholarly work products of the doctor of nursing practice: one approach to evaluating scholarship, rigour, impact and quality, Terhaar et al. (2016)	US	A total of 80 DNP projects	Descriptive study‐cohort design	To evaluate, monitor, and manage the quality of projects and work produced as evidence of scholarship upon completing Doctor of Nursing Practice education	Modification of the uncertainty, pace and complexity model	A structured framework adapted from the industry is needed to ensure that DNP programmes deliver meaningful practice and leadership outcomes DNP students lead practice‐based teams, implementing high‐quality projects that span boundaries and deliver tangible improvements This disciplined approach to education equips graduates to lead quality improvement initiatives, driving healthcare innovation and realising the DNP's full potential as the terminal nursing practice degree	Leadership Professional development Practice improvements
Leveraging Graduate Academic‐Practice Partnerships to transform health system outcomes, Wright et al. (2018)	US	DNP students (*n* = 40)	Descriptive study—cohort design	To describe an education programme for Doctorate of Nursing Practice (DNP) students in which the students, under the close supervision of academic faculty, utilise their statistical analyses and complex system coursework to study and address ‘wicked’ problems faced by healthcare organisations	SEIPS 2.0 and the ecologic model	Leveraging DNP students as consultants, the partnership reduces reliance on external experts and fosters a culture of continuous improvement DNP students gain expertise in systems, policy and strategic thinking, preparing them for leadership roles	Leadership Professional development Practice improvements
An evaluation of a virtual seminar series for Doctor of Nursing Students to promote advanced nursing practice, Lee et al. (2024)	Canada	DNP postgraduate students APNs (*n* = 11)	A pre‐experimental study	To evaluate the effectiveness of a virtual seminar series in supporting growth in clinical judgement, skills in nursing leadership and advocacy to optimise APNs' scope of practice	Clinical Instruction Evalua in Tool (CIET) Perceived confidence in APN's scope of practice scale	Virtual seminars enhance APNs' confidence and knowledge These seminars exposed diverse APN roles in clinical practice, academia, and leadership Virtual seminars to be an effective strategy for supporting newly graduated APNs' professional transitions, emphasising the importance of mentorship, experiential learning and hybrid education methods to optimise APN practice and development	Leadership Professional development Practice improvements
Predictors associated with new nursing faculty's intent to leave nursing academia: teaching preparation in doctoral program, institutional supports, and job satisfaction, Lee and Lee (2022)	US	PhD or DNP Nurses (*n* = 147)	A survey research design	To examine the relationships among demographics, doctoral teaching preparation, nurse faculty institutional support, faculty job satisfaction and intent to leave current nursing academic position in PhD‐ and DNP‐prepared faculty	The Transition to the Nurse Faculty Role Survey (TNFRS) The Faculty Needs Assessment Tool (FNAT) Faculty Members' Job Satisfaction Survey Intention to leave academic position questionnaire	Older age, holding a PhD and job dissatisfaction were significant factors influencing doctoral prepared nursing faculty's intent to leave academia. PhD‐prepared faculty, despite reporting higher levels of institutional support and job satisfaction DNP‐prepared counterparts were more likely to leave academic roles Targeted strategies are needed to retain this group. Efforts should focus on promoting job satisfaction, addressing the unique challenges PhD‐prepared faculty face, and encouraging nurses to pursue doctoral degrees earlier in their careers The need to update doctoral programme curricula to include teaching‐related courses and provide comprehensive teaching preparation for doctoral students could better equip new faculty for their roles	Career advancement Job satisfaction
Team based learning in the clinical setting: perpectives of doctor of nursing practice students, Minges et al. (2019)	US	DNP students (*n* = 16)	Descriptive cohort study	To provide direction as to how principles of team‐based learning could be applied to the ‘real‐world’ clinical setting	Class discussion	DNP students perceived that team‐based learning could be implemented in clinical settings, potentially reducing isolationism and improving interprofessional communication, job satisfaction, respect, quality of care, cost efficiency, coordination and patient safety	Job satisfaction Leadership Professional development Practice improvements
The efficacy of a Doctor of Nursing Practice Mentoring Program, Swanson et al. (2015)	US	DNP students (*n* = 49) Mentors (*n* = 7) Faculty (*n* = 9)	A survey research design	To present findings of student, mentor, and faculty perceptions regarding the knowledge, use and value of a formalised DNP mentoring programme staffed by recent DNP alumni adjunct faculty at a state university	Three 10‐question web‐based surveys, one for students, one for mentors and one for faculty	DNPgraduates encouraged to collaborate with other disciplines, assume leadership roles and serve as mentors at the clinical, educational or executive level Mentoring relationships are essential for the DNP graduate to continue growing scholarly, professionally and personally Sustaining long‐term engagement requires incentives to maintain their commitment. Initial incentives such as adjunct faculty status and library privileges could attract participation, but financial remuneration and formal contracts may be necessary to ensure a lasting mentor pool	Career advancement Professional development Leadership
Capturing the Impact of the Doctor of Nursing Practice Degree on West Texas Health Care, Boswell et al. (2019)	US	DNP‐prepared nurse leaders (*n* = 8)	Focus group qualitative study	To describe findings from a Phase 1 investigation exploring the influence of DNP‐prepared nurses in West Texas	Preplanned questions (AACN (2006) Essentials of Doctoral Education for Advanced Nursing Practice)	DNP‐prepared nurses influence health outcomes through leadership, innovation and communication, challenge processes, employ technology like simulation training, and promote evidence‐based practices for problem‐solving and policy implementation DNP graduates address systemic issues, improve efficiency and foster teamwork, while their expertise in clinical practice and education supports innovation in healthcare DNP leaders expand their impact by engaging in professional organisations, influencing policy and mentoring others	Leadership Professional development Practice improvements

## 4 Results

### 4.1 Descriptive Characteristics

The descriptive characteristics of the studies reveal that the majority were conducted in the United States (n:20), with one study originating from Canada. The studies employed diverse designs, including descriptive mixed methods (n:2), observational studies (n:2), qualitative and thematic analyses (n:3), descriptive surveys (n:7), cohort‐based approaches (n:4), pre‐post non‐experimental designs (n:2) and action research projects (n:1). The sample populations varied widely, encompassing DNP graduates and students, nurse practitioners (NPs), chief nursing executives (CNEs), doctoral‐prepared nurses, mentors and faculties (Table 1). The overarching aims of the studies were to evaluate the impact of DNP education on career advancement, leadership roles and healthcare outcomes, as well as to explore educational methodologies such as mentoring, team‐based learning and virtual seminars. Additionally, they examined job satisfaction and professional development within nursing practice. Data collection tools included online and paper‐based surveys, reflective assignments, focus group discussions, instruments such as the Nurse Practitioner‐Primary Care Organizational Climate Questionnaire (NP‐PCOCQ) and various scales measuring leadership, cultural competence and confidence (Table 1). Collectively, these studies provide a comprehensive understanding of the influence of DNP education on advancing professional roles, fostering leadership competencies and improving healthcare systems. The themes from the scoping review revealed the benefits and challenges of being a DNP graduate (Table 1).

### 4.2 Benefits of Being a DNP Graduate

#### 4.2.1 Advancing Career and Leadership Development

DNP education has been widely recognised as a foundation for advanced expertise and professional growth in nursing careers (Beeber, Johnson, and Lee 2019; Beeber, Palmer, et al. 2019). Studies indicate that DNP graduates often experience career advancements, with many securing leadership positions such as chief nursing officers and quality directors (Beeber, Johnson, and Lee 2019; Beeber, Palmer, et al. 2019; Embree et al. 2018; Giordano et al. 2021; Melander et al. 2023). Notably, a significant proportion of graduates contribute to underserved areas (28%), engage in clinical practice (52%) and take on administrative roles (21%), highlighting the diverse career opportunities afforded by DNP education (Minnick et al. 2019). DNP education has been identified as a catalyst for assuming leadership roles while offering greater flexibility in career trajectories (Giordano et al. 2021; Minnick et al. 2019). Graduates also reported a strengthened sense of professional identity and increased confidence as they assumed leadership roles during their DNP education (Beeber, Johnson, and Lee 2019; Beeber, Palmer, et al. 2019; Swanson et al. 2015). Giordano et al. (2021) emphasised that after completing DNP programmes, graduates became more effective in team management and developed a stronger sense of belonging to the nursing profession as they contributed to meaningful changes in patient care through their projects (Giordano et al. 2021).

Many graduates have participated in advanced training programmes, such as leadership academies, as part of their DNP education, further enhancing their professional skills (Fitzgerald and McNulty 2017; Robinson 2023). DNP graduates have supported organisational change processes by leading patient safety and quality improvement initiatives (Giordano et al. 2021). For example, various DNP‐led projects have successfully restructured infection control protocols, resulting in a 30% reduction in infection rates, integrated electronic health records to reduce processing times by 25%, and launched professional development programmes that increased employee engagement by 18% (Beeber, Johnson, and Lee 2019; Beeber, Palmer, et al. 2019; Prado‐Inzerillo et al. 2023). Similarly, DNP‐trained leaders have implemented mentorship and professional development programmes that increased nurse satisfaction and engagement (Lee et al. 2024; Prado‐Inzerillo et al. 2023; Swanson et al. 2015). By optimising operational workflows, graduates have contributed to significant efficiency improvements, reducing hospital costs by 10% (Beeber, Johnson, and Lee 2019; Beeber, Palmer, et al. 2019; Embree et al. 2018).

#### 4.2.2 Improving Clinical Practice and Patient Outcomes

DNP graduates have led evidence‐based practices to improve patient outcomes and enhance the quality‐of‐care processes. For example, implementing updated care guidelines has resulted in a 20% increase in patient satisfaction, while revised discharge planning processes have reduced post‐discharge readmission rates by 12% (Giardino and Hickey 2020; Melander et al. 2023). Additional efforts include developing hand hygiene protocols that reduce infection rates by 30%, updating guidelines to enhance patient safety and redesigning electronic prescription systems to streamline processes (Beeber, Johnson, and Lee 2019; Beeber, Palmer, et al. 2019; Giardino and Hickey 2020). By leading multidisciplinary teams, DNP graduates have successfully accelerated patient care processes by 25% and improved resource utilisation, reducing patient waiting times by 18% (Root et al. 2020; Melander et al. 2023). However, the success of these initiatives is often inadequately measured using concrete metrics, limiting the recognition and dissemination of their impact (Giardino and Hickey 2020).

Aside from their contribution to quality improvement in acute care settings, DNP graduates have spearheaded community‐based healthcare projects, expanding access to care in rural areas and increasing childhood vaccination rates by 25% (Root et al. 2020). Additionally, DNP‐led educational programmes have improved medication adherence rates in chronic disease management by 30% (Beeber, Johnson, and Lee 2019; Beeber, Palmer, et al. 2019). Prado‐Inzerillo et al. (2023) found that 93% of DNP graduates reported enhanced professional satisfaction from leading quality improvement initiatives and witnessing the tangible outcomes of their projects. Furthermore, graduates who developed cultural competence through their DNP training reported increased job satisfaction due to their ability to provide more inclusive and effective patient care. However, despite all these benefits, DNP graduates also noted some challenges.

### 4.3 Challenges Faced by DNP Graduates

#### 4.3.1 Recognition and Utilisation of DNP Competencies

While DNP graduates often excel in leadership and quality improvement projects, DNP graduates often face challenges in career progression due to a lack of standardised roles that explicitly require a DNP degree (Beeber, Johnson, and Lee 2019; Beeber, Palmer, et al. 2019; Lee and Lee 2022). This underrecognition limits the visibility of their contributions and creates disparities in leadership opportunities. Additionally, systemic barriers, such as inconsistent hiring criteria and organisational resistance to new leadership models, further hinder professional advancement (Embree et al. 2018; Lee and Lee 2022).

#### 4.3.2 Financial and Institutional Barriers

High educational costs remain a significant barrier, limiting graduates' flexibility in career choices (Minnick et al. 2019). Many DNP graduates experience financial strain due to tuition expenses, particularly those balancing clinical or administrative responsibilities alongside their education (Embree et al. 2018). Furthermore, the long‐term sustainability of DNP‐led community health projects is often compromised due to insufficient funding and organisational support (Beeber, Johnson, and Lee 2019; Beeber, Palmer, et al. 2019; Lee and Lee 2022). DNP graduates also encounter limited opportunities in academic and research roles, as PhD‐prepared candidates are often prioritised for faculty and research positions (Rosenfeld 2024). This restricts the ability of DNP‐trained professionals to contribute to nursing education despite their expertise in evidence‐based practice and leadership (Rosenfeld 2024). Graduates also report difficulties balancing patient care duties with new leadership responsibilities, which can negatively impact job satisfaction (Beeber, Johnson, and Lee 2019; Beeber, Palmer, et al. 2019). Systemic barriers such as role ambiguity and fragmented policies prevent DNP graduates from fully utilising their leadership and evidence‐based practice training (Lee and Lee 2022). Combined with the emotional strain of balancing clinical and leadership roles, these challenges can lead to professional burnout (Beeber, Johnson, and Lee 2019; Beeber, Palmer, et al. 2019).

## 5 Discussion

This scoping review synthesised findings from 21 studies, highlighting the multifaceted contributions of DNP programs to career advancement, job satisfaction, leadership skills and improvements in healthcare systems. DNP education emerges as a significant model that strengthens the impact of nursing in leadership, career advancement, professional satisfaction and contributions to healthcare systems (Melander et al. 2023). Findings from this scoping review revealed the critical roles DNP graduates assume in leadership positions (Terhaar et al. 2016; Wright et al. 2018; Root et al. 2020), their capacity to lead community health improvement projects (Minnick et al. 2019; Lee et al. 2024) and their success in initiatives aimed at enhancing the quality of healthcare services (Beeber, Johnson, and Lee 2019; Beeber, Palmer, et al. 2019; Lee et al. 2024). Despite their effectiveness in leadership roles, DNP graduates faced significant challenges, including career path uncertainties (Giardino and Hickey 2020; Boswell et al. 2019; Jenkins and McCauley 2021), financial burdens (Minnick et al. 2019) and a lack of academic opportunities (Rosenfeld 2024; Lee and Lee 2022). For instance, the fact that most leadership positions do not require a DNP degree often leads to the under‐recognition of graduates' competencies (Beeber, Johnson, and Lee 2019; Beeber, Palmer, et al. 2019). Additionally, the high costs of DNP programmes place financial strain on graduates, limiting their career progression (Minnick et al. 2019; Rosenfeld 2024).

However, supportive mechanisms such as mentorship (Robinson 2023) and group‐based collaboration (Burson et al. 2019) within DNP programmes highlight the strengths of this educational model. The success of community health projects, for example, demonstrates DNP graduates' contributions to addressing health disparities and improving patient care quality (Root et al. 2020; Bleich et al. 2019). Furthermore, initiatives like implementing care protocols to reduce infection rates (Beeber, Johnson, and Lee 2019; Beeber, Palmer, et al. 2019) and developing patient safety guidelines (Embree et al. 2018) underscore DNP graduates' substantial impact on healthcare systems. DNP education offers significant opportunities for nursing leadership and professional growth. Nevertheless, the need for more inclusive educational programmes has been emphasised (Burson et al. 2019). Strengthening financial support mechanisms is crucial to alleviating the economic burden faced by graduates (Minnick et al. 2019). Moreover, systematic changes are required to enhance the recognition of DNP graduates' contributions and professional roles in healthcare systems (Boswell et al. 2019). Lastly, there is a call for a broader evaluation of DNP graduates' career development and healthcare contributions to contextualise their impact better (Swanson et al.^2015^; Fitzgerald and McNulty 2017; Giardino and Hickey 2020; Jenkins and McCauley 2021; Martsolf and Auerbach 2021; Lee and Lee 2022; Prado‐Inzerillo et al. 2023; Kidd and McCauley 2024).

### 5.1 Benefits of Being a DNP Graduate

The findings of this scoping review highlight the significant role of DNP education in advancing the careers of nursing professionals. DNP graduates are shown to take on leadership roles in healthcare systems, lead organisational change projects, implement quality improvement initiatives and secure advanced administrative or clinical positions (Beeber, Johnson, and Lee 2019; Beeber, Palmer, et al. 2019; Melander et al. 2023; Minnick et al. 2019). For instance, Melander et al. (2023) reported that 76% of DNP graduates received promotions after completing their degree, transitioning into key roles such as chief nursing officers and quality directors. Similarly, Minnick et al. (2019) emphasised that DNP education provides flexibility in career pathways, enabling graduates to excel in clinical practice, education and leadership roles. These findings are consistent with existing literature, reinforcing DNP education's value in enhancing professional roles. Robinson (2023) highlighted that the leadership skills developed through DNP programs, such as systems thinking and data analysis, prepare graduates to tackle complex healthcare challenges. Additionally, Root et al. (2020) demonstrated that DNP graduates play pivotal roles in reshaping organisational practices and improving operational efficiency. These graduates also serve as role models, driving mentorship and fostering collaboration across disciplines. Furthermore, Greenfield et al. (2019) emphasised the importance of leadership academies in equipping DNP graduates with the tools to address systemic challenges effectively. However, some studies, such as Rosenfeld (2024), have pointed out that the lack of mandatory requirements for a DNP degree in specific leadership roles limits its recognition, creating barriers to career advancement. Beeber et al. (2019b) also noted that limited organisational support and insufficient mentorship opportunities could hinder the career growth of DNP graduates. Addressing these gaps requires targeted strategies, such as embedding leadership preparation into DNP curricula and fostering collaborations between academic institutions and healthcare organisations. These findings underscore the need for mentorship programmes and leadership academies to support better graduates leveraging their leadership skills. Healthcare systems must prioritise creating structured pathways that recognise the unique capabilities of DNP‐prepared nurses in leadership pipelines.

### 5.2 Leadership and Organisational Efficiency

The findings of this scoping review demonstrate that DNP education positively impacts nurses' job satisfaction. Many reviewed studies indicate that DNP graduates experience enhanced professional identity and increased job satisfaction after achieving successful outcomes in leadership projects (Giardino and Hickey 2020; Prado‐Inzerillo et al. 2023). For instance, Prado‐Inzerillo et al. (2023) reported that 93% of DNP graduates stated that their education had significantly improved their job satisfaction. These findings underscore the critical role that leadership projects play in fostering job satisfaction (Beeber, Johnson, and Lee 2019; Beeber, Palmer, et al. 2019; Robinson 2023). Furthermore, the mentorship and collaborative learning approaches integrated into DNP education are pivotal in building professional confidence. Minges et al. (2019) highlighted that group work and mentorship, often utilised during DNP education, significantly improve nurses' job satisfaction. These approaches enhance teamwork and foster a culture of continuous professional growth. However, some contradictions regarding the impact of DNP education on job satisfaction exist in the literature. For example, Beeber, Johnson, and Lee (2019) and Beeber, Palmer, et al. (2019) noted that balancing leadership responsibilities with patient care time can present challenges, sometimes negatively impacting job satisfaction. Additionally, Singleton et al. (2017) observed that while DNP education improves professional skills such as cultural competence, these improvements do not always translate into increased job satisfaction.

To bridge these gaps, there is a need for programs that integrate leadership and clinical responsibilities more effectively. Expanding longitudinal studies on DNP graduates' job satisfaction could provide deeper insights into these dynamics. The findings of this scoping review emphasise that DNP education plays a pivotal role in preparing nurses for leadership positions and enabling them to make significant contributions to healthcare systems.

DNP graduates are trained to apply systems thinking, advanced data analysis, and evidence‐based practices to address complex healthcare challenges (Robinson 2023; Root et al. 2020). For example, Root et al. (2020) highlighted that DNP‐prepared leaders excel in reshaping organisational workflows, improving operational efficiency and fostering interdisciplinary collaboration. Similarly, Robinson (2023) noted that DNP graduates use their leadership skills to lead transformative initiatives, such as mentorship programmes and organisational change projects, which improve team engagement and morale. These contributions have been further validated through quality improvement projects. Beeber et al. (2019) reported that DNP‐trained nurses spearheaded initiatives that reduced hospital‐acquired infections and streamlined discharge processes, improving patient outcomes. Additionally, Melander et al. (2023) demonstrated that DNP graduates effectively implemented interprofessional collaboration models to enhance healthcare delivery and patient satisfaction.

### 5.3 Challenges of the DNP Graduates

This scoping review revealed some challenges DNP graduates encounter in achieving career growth and satisfaction, aligning with existing evidence on systemic and structural barriers. While DNP education provides essential leadership and advanced practice skills, the lack of mandatory requirements for the degree in leadership roles often limits its recognition and utility (Rosenfeld 2024; Beeber, Johnson, and Lee 2019; Beeber, Palmer, et al. 2019). For instance, Rosenfeld (2024) highlighted that DNP graduates are underrepresented in research and academic roles despite their significant contributions to clinical practice and leadership. Similarly, Beeber, Johnson, and Lee (2019) and Beeber, Palmer, et al. (2019) emphasised that the absence of clear career pathways for DNP‐prepared nurses restricts their professional progression, underscoring the need for more structured organisational strategies. Financial barriers also emerged as a significant obstacle. Minnick et al. (2019) noted that the high cost of DNP education limits career flexibility for many graduates, as they may prioritise financial stability over professional opportunities. This is consistent with findings from Embree et al. (2018), who pointed out that DNP‐prepared nurses often lack institutional support for advanced training or leadership roles. These financial and institutional limitations restrict professional advancement and reduce the sustainability of community health projects and quality improvement initiatives led by DNP graduates (Beeber, Johnson, and Lee 2019; Beeber, Palmer, et al. 2019). Another challenge lies in managing the dual responsibilities of leadership and clinical practice. Beeber et al. (2019) observed that DNP‐prepared nurses often struggle to balance competing priorities, such as leading organisational initiatives while maintaining clinical practice. This dual burden can negatively impact both their job satisfaction and professional identity. These findings align with broader literature highlighting the emotional and professional strain caused by role ambiguity and fragmented organisational support (Swanson et al. 2015; Embree et al. 2018). Overall, the challenges identified in this review reflect systemic barriers that limit the full potential of DNP graduates. Existing literature emphasises that addressing these challenges requires institutional support, mentorship opportunities, and role standardisation to enable DNP graduates to effectively utilise their advanced training and contribute to healthcare systems (Rosenfeld 2024; Minnick et al. 2019). Without these structural improvements, DNP graduates may encounter limited recognition and underutilisation of their competencies.

### 5.4 Strategies and Resources to Enhance Career Satisfaction and Professional Outcomes for DNP‐Prepared Nurses

The findings of this scoping review highlight several strategies and resources that can enhance the career satisfaction and professional outcomes of DNP‐prepared nurses. Mentorship programmes emerged as a critical factor in fostering professional growth and supporting DNP graduates transitioning into leadership roles (Robinson 2023; Swanson et al. 2015). For example, Swanson et al. (2015) demonstrated that formalised mentoring relationships enhance career satisfaction and facilitate the continuous professional development of DNP graduates in clinical, educational and executive settings. Leadership academies and advanced training programmes were also identified as valuable resources. Robinson (2023) reported that participation in such programs equips DNP graduates with the tools to address complex organisational challenges, fostering confidence and strategic decision‐making skills. Similarly, Greenfield et al. (2019) noted that leadership‐focused continuing education programs improve DNP graduates' ability to navigate healthcare systems and implement quality improvement initiatives effectively.

Institutional support and funding opportunities play a crucial role in maximising the professional outcomes of DNP graduates. Minnick et al. (2019) highlighted the importance of organisational investment in leadership development programmes and evidence‐based practice initiatives, which enable graduates to apply their advanced skills more effectively. Embree et al. (2018) also emphasised the need for financial support mechanisms, such as scholarships or grants, to alleviate the economic burden of DNP education and empower graduates to pursue career advancement opportunities. However, challenges remain in implementing these strategies effectively. Beeber et al. (2019b) noted that limited access to structured mentorship and leadership resources often hinders the full utilisation of DNP graduates' skills. Furthermore, Rosenfeld (2024) underscored the need for clearer career pathways and better‐define roles to ensure DNP graduates transition seamlessly into leadership and administrative positions. To address these challenges, healthcare organisations and academic institutions must collaborate to create structured support systems for DNP‐prepared nurses. This includes expanding mentorship programs, providing financial incentives, and offering tailored leadership training to meet the unique needs of DNP graduates. Future research should explore the long‐term impact of these strategies on DNP graduates' career satisfaction and professional contributions. Additionally, policymakers should advocate for greater institutional recognition of the DNP degree to ensure graduates can access roles aligned with their advanced training and expertise.

### 5.5 Limitation

This scoping review has several limitations that warrant consideration. First, the review exclusively focused on studies published in English, which may have excluded relevant research published in other languages, limiting the global applicability of the findings. Second, the inclusion criteria were restricted to peer‐reviewed articles, potentially omitting valuable insights from grey literature, dissertations and organisational reports. The reliance on indexed databases may have excluded relevant studies unavailable in these sources. Third, the variability in study designs, populations and methodologies among the included studies presented challenges in synthesising findings comprehensively. Differences in data collection tools and outcome measures further complicated cross‐study comparisons and limited the generalisability of conclusions. Fourth, the predominance of US‐based studies in this review reduces the applicability of findings to healthcare systems and cultural contexts outside the United States. Lastly, while thematic analysis ensured a systematic approach to identifying key patterns, the subjective nature of qualitative synthesis may have introduced interpretive bias. Additionally, inherent limitations of scoping reviews, such as the lack of critical appraisal of study quality, may have influenced the conclusions' robustness. Future research should address these limitations by including studies in multiple languages, exploring diverse healthcare settings and populations and incorporating grey literature. Employing systematic reviews with meta‐analyses could strengthen the evidence base by providing a more critical and quantitative synthesis of findings.

## 6 Conclusion

This review comprehensively explored the impact of DNP education on nurses' career advancement, job satisfaction, leadership competencies and contributions to healthcare systems. The findings underscore that DNP education supports individual growth by enhancing leadership roles and equips nurses with the skills to drive systemic improvements in healthcare. DNP graduates contribute directly to improved health outcomes and organisational efficiency through quality improvement projects, evidence‐based practices and patient safety initiatives. Furthermore, cultural proficiency and interdisciplinary collaboration empower nurses to address healthcare disparities and deliver inclusive care to diverse populations, strengthening public health outcomes. However, despite its transformative potential, DNP education faces challenges that must be addressed to maximise its impact. Graduates encounter career path uncertainties, high educational costs, and insufficient recognition of their competencies. Strengthening mentorship opportunities, expanding financial support mechanisms and promoting institutional recognition of DNP‐prepared nurses are critical steps to overcoming these barriers. Moreover, DNP programmes should prioritise financial literacy and advanced leadership training to prepare graduates for strategic roles within healthcare systems. By addressing these challenges, stakeholders, including policymakers, academic institutions and healthcare organisations, can unlock the full potential of DNP education to shape the future of nursing. This review highlights the transformative role of DNP graduates in driving innovation, reducing disparities and fostering sustainable improvements in healthcare delivery. As healthcare systems face increasingly complex challenges, DNP‐prepared nurses are well positioned to create a more equitable, efficient and patient‐centred future.

## Author Contributions

**Tuba Sengul:** writing – review and editing, writing – original draft, project administration, methodology, investigation, formal analysis, conceptualization. **Seda Sarikose:** writing – review and editing, writing – original draft, formal analysis, data curation, conceptualization, methodology. **Violeta Lopez:** writing – review and editing, writing – original draft, conceptualization, methodology. **Holly Kirkland‐Kyhn:** writing – review and editing, writing – original draft, conceptualization, methodology.

## Ethics Statement

Ethical approval for this study was not required because it involved neither experimentation nor patient involvement in active data collection.

## Conflicts of Interest

The authors declare no conflicts of interest.

## Supporting information


Data S1.

## Data Availability

Data sharing is not applicable to this article as no datasets were generated or analyzed during the current study.
